# Supplementary material for: Active Field Theory Approach to Explain Size Control of Transcriptional Condensates
Source: Macromol Biosci. 2026 Oct 7;26(10):e70268. doi: 10.1002/mabi.70268 (PMC13643503; doi:10.1002/mabi.70268)
Supplement: Supplementary file 1 [file MABI-26-e70268-s001.pdf]

Supplemental figures for

Active field theory approach to explain size control of transcriptional condensates

Kathrin Hertäg,[1] Samuel Shoup,[2,3] Leonhard T. Thews,[4] Radhika Khatter,[4] Eugenio Ferrario,[5] Joshua F. Robinson,[6,7] Sina Wittmann,[4,8] Sandra Schick,[2,8] and Thomas Speck[1,*]

[1]*Institute for Theoretical Physics IV, University of Stuttgart, Heisenbergstr. 3, 70569 Stuttgart, Germany*
[2]*Chromatin Regulation Group, Institute of Molecular Biology (IMB), Mainz, Germany*
[3]*Max Planck Graduate Center (MPGC), Mainz, Germany*
[4]*Protein Disorder in Transcription Group, Institute of Molecular Biology (IMB), Mainz, Germany*
[5]*Protein Production Core Facility, Institute of Molecular Biology (IMB), Mainz, Germany*
[6]*STFC Hartree Centre, Sci-Tech Daresbury, Warrington, WA4 4AD, United Kingdom*
[7]*H. H. Wills Physics Laboratory, University of Bristol, Bristol BS8 1TL, United Kingdom*
[8]*Institute for Quantitative and Computational Biosciences (IQCB), Johannes Gutenberg University Mainz, Germany*

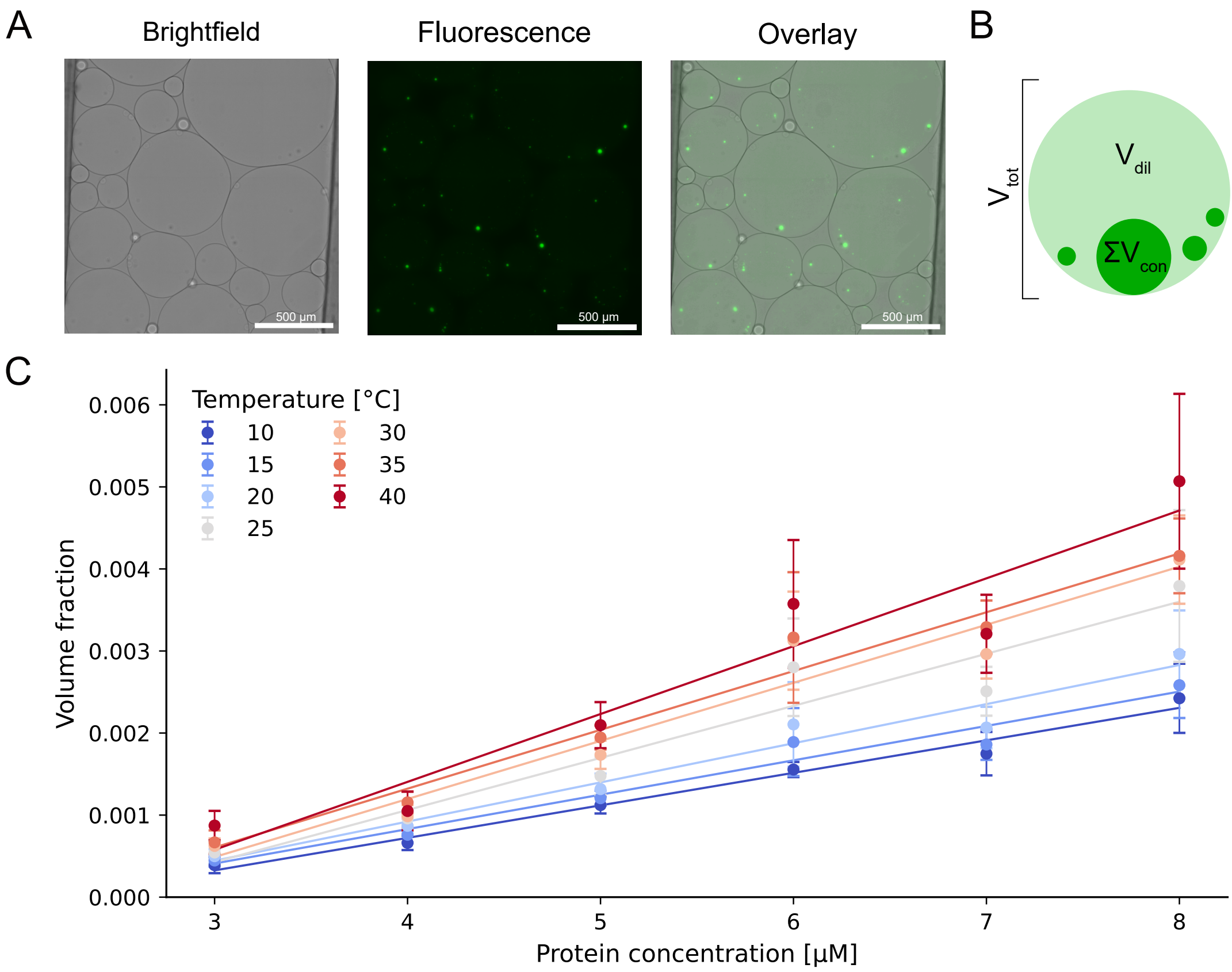

SUPP. FIGURE 1 | Determination of dense-phase volume fractions using the inPhase assay. (A) Representative microscopy images of BRD4-L emulsions acquired on a temperature-controlled stage, shown as brightfield, fluorescence and overlay (brightfield + fluorescence channels). Fluorescence highlights condensates within individual emulsion droplets (scale bars, 500 µm). (B) Schematic illustrating quantification of the dense-phase volume fraction. For each emulsion droplet, the total droplet volume (V_{tot}) was estimated from droplet geometry and the cumulative condensate volume (V_{con}) was obtained from segmented fluorescence signal. The volume fraction was calculated as $\phi = V_{\mathrm{con}}/V_{\mathrm{tot}}$. (C) Dense-phase volume fraction as a function of total protein concentration for temperatures between 10 °C and 40 °C. Points represent averages of individual droplets, scale bars standard deviation of the mean; solid lines denote linear fits at each temperature.

* thomas.speck@itp4.uni-stuttgart.de

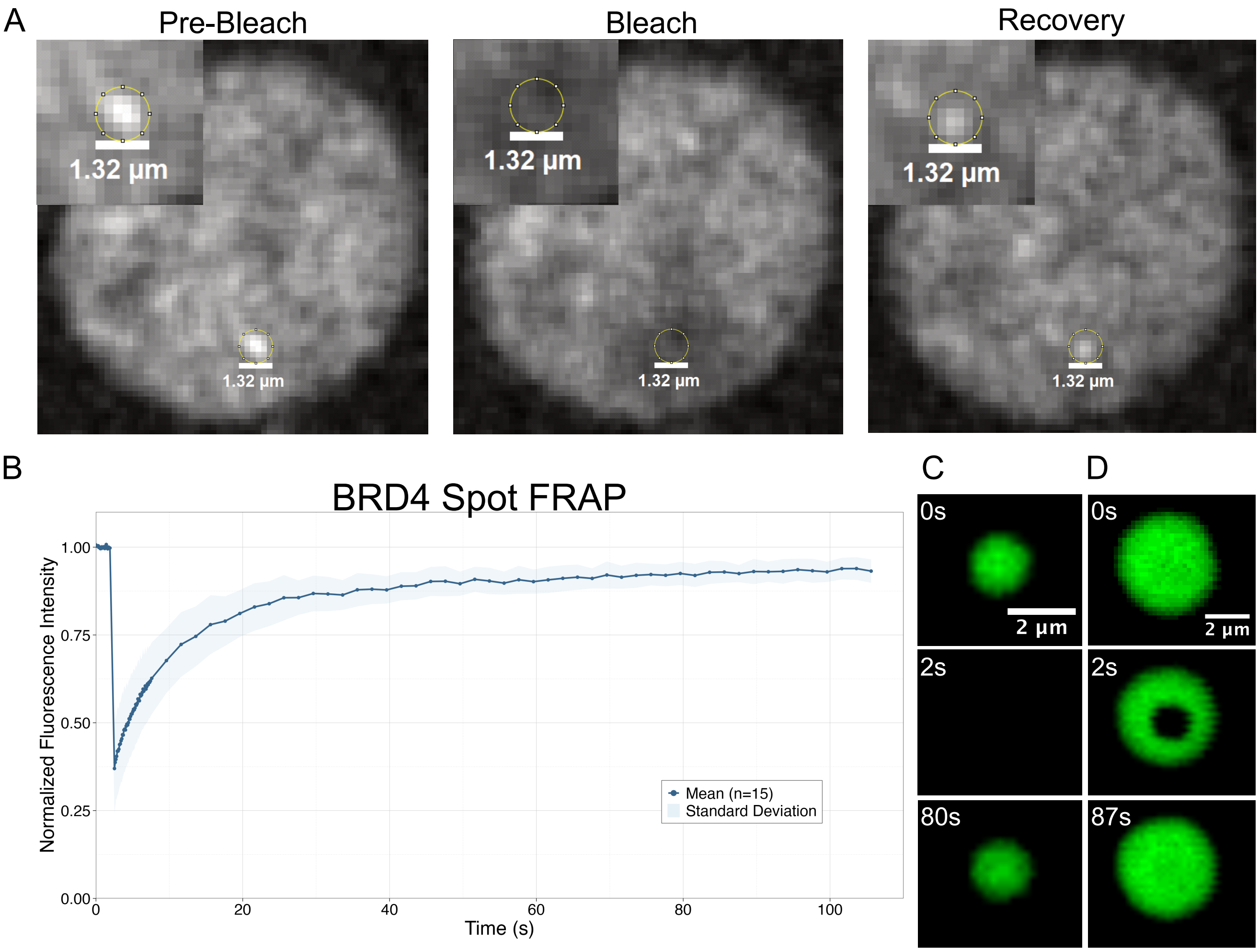

SUPP. FIGURE 2 | BRD4::eGFP undergoes rapid fluorescence recovery after photobleaching. (A) Representative fluorescence recovery after photobleaching (FRAP) experiment performed on BRD4::eGFP in live HAP1 cells. Confocal images show the bleached region (dashed circle, ∅1.32 µm [6 pixel]) at the pre-bleach (0 s), bleach (+3.2 s), and recovery (+43 s) time points. (B) Quantification of normalized fluorescence recovery after photobleaching in droplets ($n = 15$) in vitro. Data are represented as mean ±SD in shaded region. (C) Representative images show recovery of fully bleached droplets and (D) spot bleached droplets (ROI diameter approx. 1.5 µm).

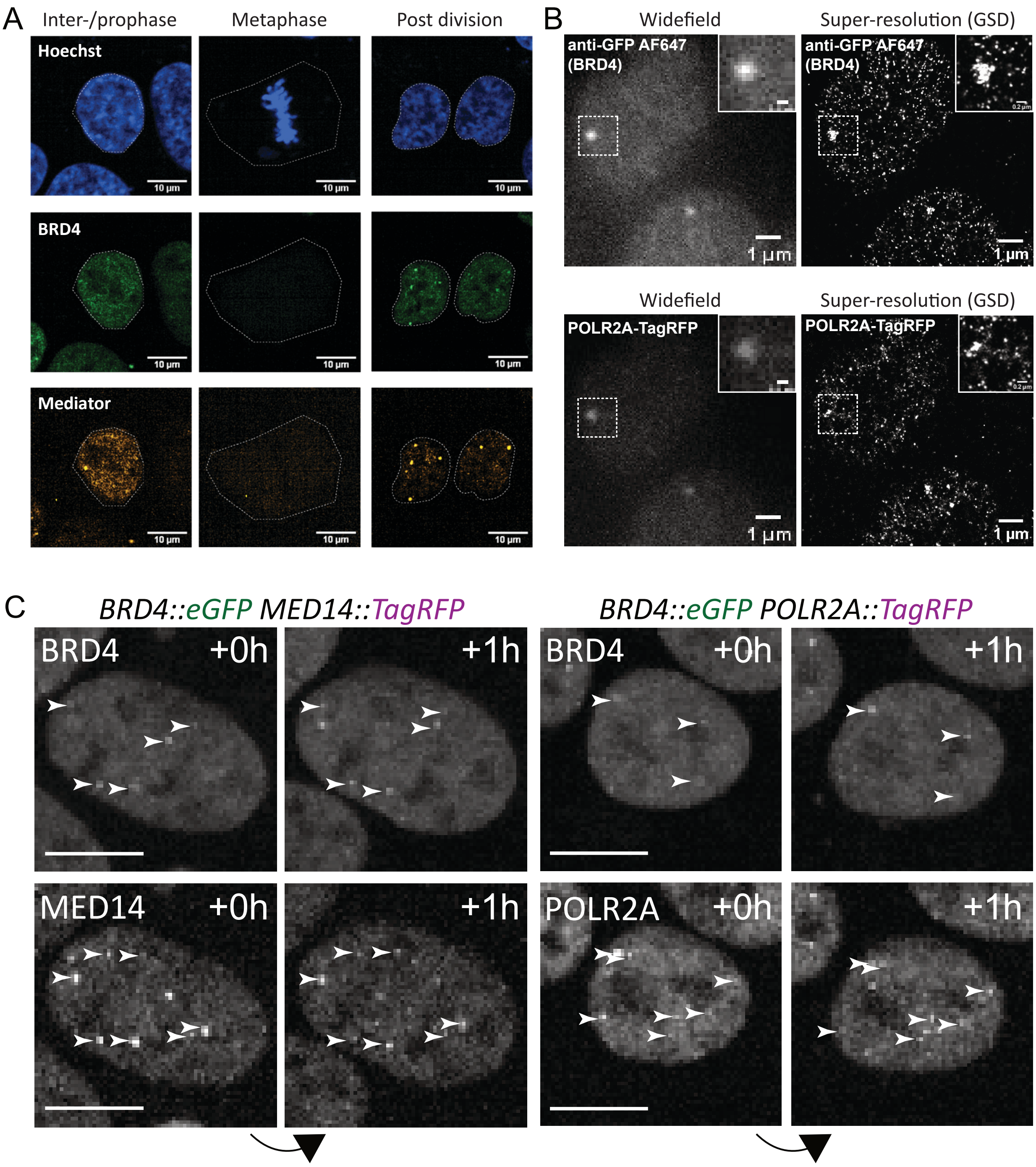

SUPP. FIGURE 3 | BRD4 and Mediator condensates are dissolved during mitosis and resolved by super-resolution microscopy. (A) Representative live-cell fluorescence microscopy images of HAP1 BRD4::eGFP MED14::TagRFP cells across cell cycle stages. Hoechst (DNA), BRD4, and Mediator (MED14) channels are shown at inter-/prophase, metaphase, and post-division time points (scale bar, 10 µm), illustrating the mitotic dissolution and re-establishment of transcriptional condensates following cell division. (B) Comparison of widefield and super-resolution ground-state depletion (GSD) microscopy images of BRD4 and POLR2A in HAP1 cells. BRD4 was visualized using an anti-GFP::AF647 antibody; POLR2A was imaged via TagRFP. Single-molecule localization processing was applied to generate super-resolution images (scale bar, 1 µm), revealing the sub-diffraction structure of individual BRD4 and POLR2A foci. (C) Time-lapse fluorescence images of BRD4::eGFP MED14::TagRFP (left) and BRD4::eGFP POLR2A::TagRFP (right) HAP1 cells at 0 h and +1 h. Arrowheads indicate BRD4/MED14 and BRD4/POLR2A spots that are visible in both timepoints. Images demonstrate the stability and persistence of transcriptional condensate over a one-hour imaging interval.
